# Advances in *Polygonatum sibiricum* polysaccharides: Extraction, purification, structure, biosynthesis, and bioactivity

**DOI:** 10.3389/fnut.2022.1074671

**Published:** 2022-12-05

**Authors:** Dan Liu, Wei Tang, Chao Han, Shaoping Nie

**Affiliations:** ^1^Key Laboratory of Pollution Exposure and Health Intervention of Zhejiang Province, College of Biology and Environmental Engineering, Zhejiang Shuren University, Hangzhou, China; ^2^College of Food Science and Technology, Zhejiang University of Technology, Hangzhou, China; ^3^State Key Laboratory of Food Science and Technology, China-Canada Joint Lab of Food Science and Technology (Nanchang), Nanchang University, Nanchang, China

**Keywords:** *Polygonatum sibiricum* polysaccharide, extraction, purification, structure, biosynthesis, bioactivity

## Abstract

*Polygonatum sibiricum* has been used as food and medicine for thousands of years, and *P. sibiricum* polysaccharides (PSPs) have become the hot research spot due to their various health-promoting functions. Numerous studies have shown that PSPs possess huge potential in the application of functional food and medicine fields. However, the research status and features of the preparation process, molecular structure, and bioactivities of PSPs are unclear. Therefore, this review makes a comprehensive summary and proposes new insights and guidelines for the extraction, purification, structural features, biosynthesis, and multiple bioactivities of PSPs. Notably, it is concluded that PSPs mainly contain several types of polysaccharides, including fructan, pectin, galactomannan, glucomannans, arabinogalactan, and galactan, and multiple bioactivates, including osteogenic activity, anti-obesity, anti-diabetes, anti-depression, antioxidant, antiglycation, and protective effect against neurotoxicity and gut microbiota regulating activity. This review contributes to the structure–function study and resource utilization of *P. sibiricum* and its polysaccharides in food fields.

## Graphical Abstract

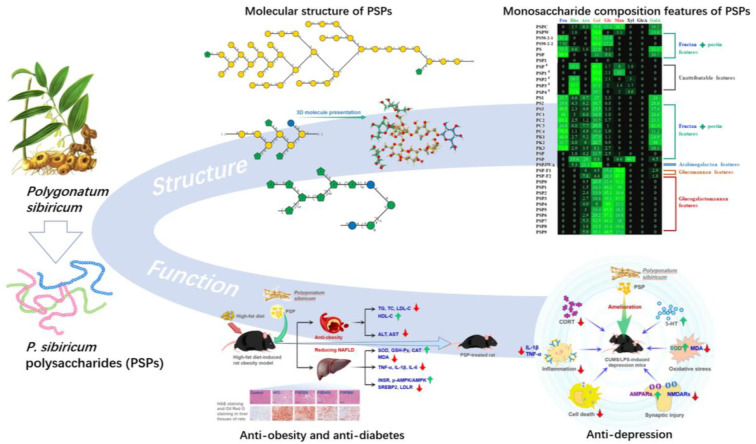

## Highlights

- New insights into the preparation and structural features of PSPs are outlined.
- PSPs contain fructan, pectin, glucogalactomannan, glucomannan, and arabinogalactan.
- PSPs biosynthesis pathway is described and discussed.
- Multiple bioactivities and action mechanisms of PSPs are summarized.
- Future perspectives are proposed to provide some new insights into PSPs study.

## Introduction

**Figure 1:**
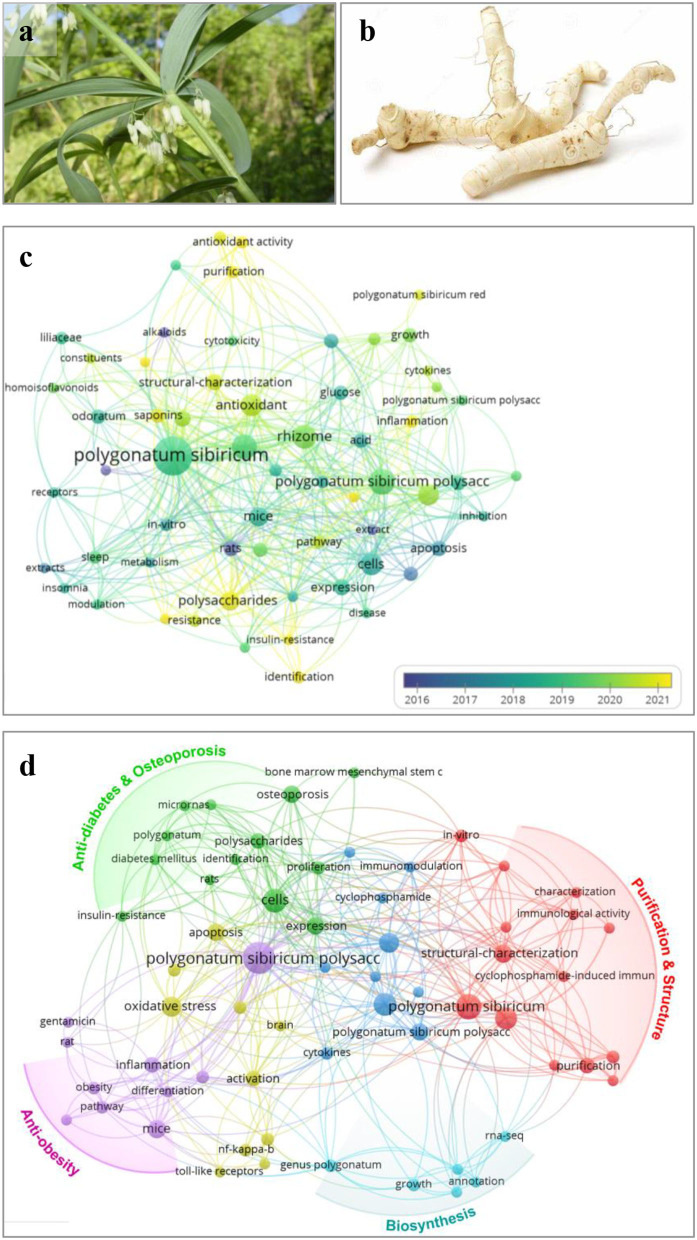
Photos of aerial part **(a)** and rhizome **(b)** of *Polygonatum sibiricum* and bibliometric analysis **(c,d)** based on papers related to *P. sibiricum* and its polysaccharides. Bibliometric analysis of *Polygonate sibiricum* research based on papers published on Web of Science core collection with the topic of “*Polygonatum sibiricum*”/“*Polygonatum*” without specific year-published restriction up to 28/09/2022. **(c)** Map of co-occurrence network of keywords based on retrieval of “*Polygonatum sibiricum*.” **(d)** Map of co-occurrence network of author keywords based on retrieval of “*Polygonatum sibiricum* AND polysaccharide*”.

*Polygonatum sibiricum* is one species belonging to the *Polygonatum* genus, and it previously belonged to the *Liliaceae* family and later the *Asparagaceae* family according to the Angiosperm Phylogeny Group system (1). Around 79 species of *Polygonatum* are recorded worldwide, and they are mainly distributed in the temperate Northern Hemisphere (2). Thereinto, 39 species are distributed in China which is the country with the most *Polygonatum* (3). *Polygonatum* has already been listed as an edible and medicinal plant in China since 2002 (4). The rhizomes of *P. sibiricum* together with *P. cyrtonema* and *P. kingianum* are known as “Huangjing” (4) (Figures 1a,b) (5), and as tonic herbs, they have been used as traditional Chinese medicine for more than a thousand years (3, 6, 7).

Based on the retrieval results from the Web of Science core collection focusing on the *P. sibiricum* and *P. sibiricum* polysaccharide, their co-occurrence network of keywords are presented in Figures 1c,d through VOSviewer 1.6.11 software analysis (8, 9). The bibliometric analysis of *P. sibiricum* research indicates that phytochemicals, including polysaccharides, saponins, alkaloids, and flavonoids, are the main research contents of *P. sibiricum* (10). Furthermore, it is seen that the research emphasis of *P. sibiricum* have turned to the “polysaccharide” in 2016–2020 as shown in Figure 1c, suggesting that *P. sibiricum* polysaccharides (PSPs) have become the hot spot in recent 5 years.

Physicochemical and biological investigations have demonstrated that polysaccharides are one of the primary bioactive compounds in *P. sibiricum* rhizome responsible for its multiple bioactivities and healthy functions. As shown in Figure 1d, studies of PSPs mainly include purification, structural characterization, and various bioactivity research. Moreover, polysaccharides have been used as the evaluation marker in quality control of “Huangjing” and its “Yuzhu” (*P. odoratum*, another *Polygonatum* genus plant) (4), particularly, Zhao et al. (5) revealed that the amounts of GalA and Gal could be the important distinguishing feature between “Huangjing” and “Yuzhu.” It has been found that PSPs at least have five types of polysaccharides including fructan (2, 5, 11), pectin (2, 5, 11), glucogalactomannan (12), glucomannans (13), and arabinogalactan-type polysaccharides (14), and the pectin and fructan are the most reported polysaccharides of PSPs. On the other hand, PSPs have been explored to possess osteogenic activity (15–17), anti-obesity (18), anti-diabetes (19), antioxidant (20, 21), antidepression (21), and so on.

As natural biological macromolecules derived from plants, PSPs have attracted increasing attention due to their potential applications in functional foods and medicine fields. Nevertheless, the research status and features of the preparation process, molecular structure, and bioactivities of PSPs are unclear. Given the importance of polysaccharides in these studies of *P. sibiricum*, this review comprehensively summarized and discussed the recent advances in the extraction, purification, structural features, biosynthesis, and bioactivities of PSPs, and sheds light on these study characteristics. This paper aims to provide the evaluable reference and information for further study of *P. sibiricum* and polysaccharides therein.

## Extraction and purification

The chemical composition of *P. sibiricum* varies greatly (Table 1). This diversity could be attributed to differences in maturity, geographic location, environmental circumstances, and extraction methods and analytical procedures in different research. Practically, extraction and purification are indispensable for the physicochemical, structural, and bioactive study. And the extraction procedure is also one part of the purification since those different polysaccharides will leach under different extraction conditions (28).

**Table 1:** Chemical composition of *Polygonatum sibiricum* polysaccharide.

**No**.	**Name**	**Extraction and purification**	**Yield (%)**	**Mw (kDa) ^a^**	**Monosaccharide composition (% molar ratio)**									**Glycosidic bonds and structural features**	**Reference**
					**Fru**	**Rha**	**Ara**	**Gal**	**Glc**	**Man**	**Xyl**	**GlcA**	**GalA**		
**1**	PSPC	Extraction: 80 °C, 1:12 (g/mL), 2 h, twice; Purification: Sevag method, dialysis (3.0 kDa)	6.2	4.0	–	2.7	6.3	29.6	15.1	36.1	–	–	10.2		Sun et al. (22)
**2**	PSPW		4.9	14.2	–	1.8	tr.	78.8	tr.	5.5	–	–	13.8		Sun et al. (22)
**3**	PS50-2-1	Extraction: 80 °C, 1:10 (g/mL), 3 h; Purification: DEAE-52-cellulose column, Sephacryl S-100 column	0.7	7.7	31.2	–	–	53.2	15.6	–	–	–	–	β-D-Glc*p*-(1 → , → 2)-β-D-Gal*p*-(1 → , → 2,6)-β-D-Gal*p*-(1 → , β-D-Fru*f*-(2 →	Liu et al. (15)
**4**	PS50-2-2		0.9	7.0	7.92	–	–	64.8	27.2	–	–	–	–	β-D-Glc*p*-(1 → , β-D-Gal*p*-(1 → , → 2)-β-D-Gal*p*-(1 → , → 6)-β-D-Gal*p*-(1 → , → 2,6)-β-D-Gal*p*-(1 → , β-D-Fru*f*-(2 →	Liu et al. (15)
**5**	PS	Extraction: 100 °C, 2 h, three times; Purification: Sevag method, dialysis (3.5 kDa)	–	630.0	33.3	6.6	1.6	22.9	3.5	–	–	–	32.1	Fructan & pectin ^c^	Zhao et al. (2)
**6**	PSP	Extraction: 80 °C, 1:30 (g/mL), 2 h, twice; Purification: α-amylase, Sevag method, dialysis (3.5 kDa)	15.4	10.0	68.9	–	–	11.0	9.4	–	–	–	10.7	→ 1)-β-D-Fru*f*-(2 → , β-D-Fru*f*-(2 → , → 1,6)-β-D-Fru*f*-(2 → , → 6)-β-D-Fru*f*-(2 → , may be with triple-helical conformations at low alkaline concentrations	Wang et al. (23)
**7**	PSP1	Extraction: water extraction (no detail); Purification: DEAE-52-cellulose column, Sephadex G150	–	322.3	–	–	–	–	–	–	–	–	–	Main chain: → 1)-β-D-Fru*f*-(2 → 1)-β-D-Fru*f*-(2 → , → 1,6)-β-D-Fru*f*-(2 → 1)-β-D-Fru*f*-(2 → ; Mian side chain: β-D-Fru*f*-(2 → , β-D-Fru*f*-(2 → 6)-β-D-Fru*f*-(2 →	Wang et al. (23)
**8**	PSP ^d^	Extraction: water extraction (no detail); Purification: Sevag reagent, activated carbon	4.4	–	–	25.1	–	63.5	1.7	8.0	1.6	–	–		Liu et al. (24)
**9**	PSP1 ^d^	Extraction: water extraction (no detail); Purification: DEAE-Sepharose Fast Flow column	–	4.4	–	–	–	82.9	2.1	15.0	–	–	–		Wang et al. (25)
**10**	PSP2 ^d^		–	2.2	–	20.5	–	74.4	2.1	–	3.0	–	–		Wang et al. (25)
**11**	PSP3 ^d^		–	7.7	–	57.7	–	37.2	2.0	1.4	1.7	–	–		Wang et al. (25)
**12**	PSP4 ^d^		–	6.5	–	72.6	–	20.7	–	2.0	4.6	–	–		Wang et al. (25)
**13**	PS1	Extraction: 100 °C, 2 h, three times; Purification: Sevag method, dialysis (3.5 kDa)	1.2	1,100.0	33.7	4.6	9.7 ^b^	27.0	1.3	–	^b^	–	24.0	DE of 41.0%, fructan & pectin ^c^	Zhao et al. (5)
**14**	PS2		1.3	1,500.0	29.8	4.3	6.2 ^b^	30.7	0.8	–	^b^	–	28.6	DE of 53.3%, fructan & pectin ^c^	Zhao et al. (5)
**15**	PS3		1.7	740.0	48.8	2.3	4.6 ^b^	25.5	1.3	–	^b^	–	17.4	DE of 40.5%, fructan & pectin ^c^	Zhao et al. (5)
**16**	PC1		1.2	2,000.0	48.0	3.0	6.6 ^b^	16.9	1.8	–	^b^	–	24.6	DE of 51.4%, fructan & pectin ^c^	Zhao et al. (5)
**17**	PC2		2.2	1,200.0	63.1	2.5	1.2 ^b^	10.5	1.7	–	^b^	–	21.1	DE of 40.7%, fructan & pectin ^c^	Zhao et al. (5)
**18**	PC3		1.4	1,200.0	24.6	4.8	7.7 ^b^	20.5	1.0	–	^b^	–	41.4	DE of 44.8%, fructan & pectin ^c^	Zhao et al. (5)
**19**	PC4		1.8	4,400.0	70.3	1.1	4.9 ^b^	10.6	1.9	–	^b^	–	11.2	DE of 49.7%, fructan & pectin ^c^	Zhao et al. (5)
**20**	PK1		0.9	1,200.0	48.9	2.7	5.2 ^b^	17.3	1.1	–	^b^	–	24.9	DE of 46.9%, fructan & pectin ^c^	Zhao et al. (5)
**21**	PK2		0.8	1,400.0	31.7	4.8	6.0 ^b^	20.7	0.9	–	^b^	–	36.0	DE of 59.2%, fructan & pectin ^c^	Zhao et al. (5)
**22**	PK3		1.0	890.0	72.8	2.9	3.5 ^b^	8.1	2.7	–	^b^	–	10.1	DE of 41.4%, fructan & pectin ^c^	Zhao et al. (5)
**23**	PSP	Extraction: water extraction, 1 h, three times; Purification: Sevag method, dialysis	–	95.1	–	1.8	4.2	11.7	2.5	1.0	–	–	–	–	Chen et al. (26)
**24**	PSP	Extraction: water extraction, 90°C, 3 h, three times; Purification: 60% ethanol precipitation, macroporous resin for decoloring, DEAE cellulose elution, and dialysis	–	–	–	15.4	24.0	8.8		6.6	40.7	–	4.5	–	Luo et al. (27)
**25**	PSPJWA	Extraction: 100 °C, 1:10 (g/mL), 3 h, twice; Purification: DEAE-cellulose column, then Sephadex G-200 column	–	–	–	1.0	4.0	14.0	–	–	–	–	–	Arabinogalactan; glycosidic bongs: Gal*p*-(1 → (7.8%), → 4)-Gal*p*-(1 → (59.2%), → 3)-Gal*p*-(1 → (1.1%), → 6)-Gal*p*-(1 → (0.7%), → 3,4)-Gal*p*-(1 → (1.7%), → 4,6)-Gal*p*-(1 → (6.2%), → 3,6)-Gal*p*-(1 → (2.7%),	Li et al. (14)
												Ara*f*-(1 → (6.6%), → 5)-Ara*f*-(1 → (7.4%), → 3,5)-Ara*f*-(1 → (5.0%), → 2,4)-Rha*p*-(1 → (2.1%),	
**26**	PSP-F1 ^e^	Extraction: water extraction, 100°C, 2 h, twice; Purification: 70% ethanol precipitation, DEAE-Sepharose fast flow column elution, and dialysis	3.4	103.0	–	–	4.0	4.5	15.2	76.3	–	–	2.9 ^e^	Glucomannan; glycosidic bongs: → 4)-Man-(1 → (68.2%), → 4)-Glc-(1 → (11.7%), → 4,6)-Man-(1 → (8.1%), → 5)-Ara-(1 → (4.0%), Glc-(1 → (3.5%), Gal-(1 → (4.5%)	Yelithao et al. (13)
**27**	PSP-F2		2.5	628.0	–	–	7.6	4.4	20.3	67.7	–	–	1.8 ^e^	Glucomannan	Yelithao et al. (13)
**28**	PSP0	Extraction: rhizomes was processed by 9 times repeated steaming with autoclave (121°C, 0.12 MPa,60 min); then ultrasonic extraction (40 kHz, 100 W) for 75 min at 60°C; Purification: papain digestion for deprotein, DEAE-cellulose column, then Sephadex G-100 column	–	6.1	–	–	0.5	26.9	21.4	51.2	–	–	–		Li et al. (20)
**29**	PSP1		–	10.3	–	–	1.5	14.3	40.2	44.0	–	–	–		Li et al. (20)
**30**	PSP2		–	4.6	–	–	2.4	13.9	45.1	38.6	–	–	–		Li et al. (20)
**31**	PSP3		–	4.4	–	–	2.7	10.8	39.1	47.5	–	–	–		Li et al. (20)
**32**	PSP4		–	5.1	–	–	4.9	9.0	64.0	22.2	–	–	–		Li et al. (20)
**33**	PSP5		–	44.3	–	–	3.0	13.4	65.5	18.2	–	–	–		Li et al. (20)
**34**	PSP6		–	75.3	–	–	2.9	20.2	57.1	19.8	–	–	–		Li et al. (20)
**35**	PSP7		–	64.4	–	–	5.3	32.5	44.2	18.0	–	–	–		Li et al. (20)
**36**	PSP8		–	44.5	–	–	3.8	33.5	33.4	29.4	–	–	–		Li et al. (20)
**37**	PSP9		–	57.5	–	–	5.9	30.1	46.5	17.5	–	–	–		Li et al. (20)

a : the molecular weight of polysaccharides is based on the main peaks (area ratio is close to or more than 60%) of HPGPC, etc.

b : (PSP 13–22) the molar ratio of Ara/Xyl here may be the molar ratio of Ara or Xyl or their mixture for that the methods of HPLC-RID and pre-column 1-phenyl-3-methyl-5-pyrazolone derivatization HPLC cannot separate the signals of Ara and Xyl well.

c : (PSP 13–22) fructan is with β-2,1- and β-2,6-D-fructosidic linkages; pectin is with α-1,4-D-galactosiduronic linkages.

d : (PSP 8–12) the determination of uronic acids (GalA and GlcA) was not mentioned in original papers, and the monosaccharide composition data were possibly calculated except for the uronic acid.

e : (PSP 26-27) here the amount of GalA can be that of GlcA since the original paper only provided a uronic acid amount without further identification. PS1, wild *P. sibiricum* from Shangluo, Shaanxi; PS2, wild *P. sibiricum* from Hanzhong, Shaanxi; PS3, wild *P. sibiricum* from Shiyan, Hubei; PS4 was not considered here due to that its main Mw (5.4 kDa) is too small, which might not belong to polysaccharide through HPGPC method. PC1, wild *P. cyrtonema* from Yichun, Jiangxi; PC2 wild *P. cyrtonema* from Qingyuan, Guangdong; PC3, wild *P. cyrtonema* from Lu'an, Anhui; PC4, cultivated *P. cyrtonema* from Chizhou, Anhui; PK1, wild *P. kingianum* from Yongde County, Yunnan; PK2 wild *P. kingianum* from Yun County, Yunnan; PK3, cultivated *P. kingianum* from Honghe, Yunnan; DE, degree of esterification.

**Other:** -: no record.

As shown in Table 1, the hot-water extraction method is the most used for PSPs preparation. The common temperature, time, and ratio of liquid-to-solid range from 80 to 100°C, 2 to 3 h, and 1:(10–30) g/mL, respectively. Overall, PSPs are generally extracted by traditional hot-water extraction methods; therefore, the extraction efficiency of PSPs using novel techniques, such as ultrasonic, microwave, and subcritical extraction, should be further uncovered. Notably, the yield of PSPs has a different calculation basis, which depends on the purification procedures. It should be noted that PSPs with yields of 4.4–15.4% are those purified roughly by the Sevag method for deprotein, while those with much lower yields of 0.7–2.2% are generally subjected to finer purification, for instance, the column chromatography method.

**Figure 2:**
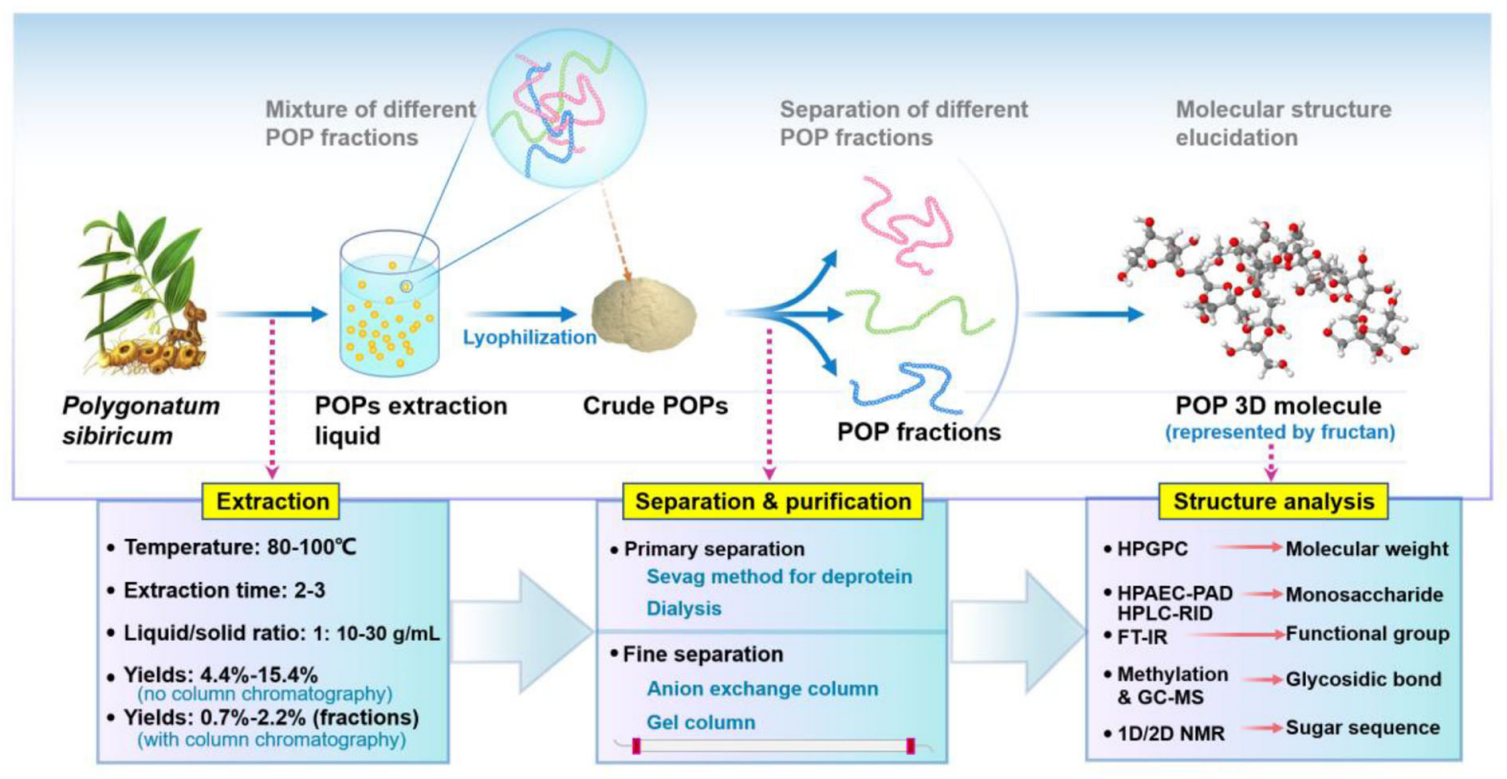
Overall presentation of extraction, purification, and structure analysis of PSPs.

As shown in Figure 2, the primary separation procedures of PSPs include the Sevag method and dialysis. Using the Sevag method will also empirically decrease the yield particularly when its repeating time increases, since polysaccharides may co-precipitate with denatured protein (29). The enzymatic hydrolysis method generally has higher efficiency, more simple operation, and a lower loss rate of polysaccharides (28, 30, 31), which is recommended to be applied or combined with the Sevag method without repetition in removing protein of PSPs. As for the finer separation by column chromatography, the anion exchange column combined with the gel column is the most used method for PSPs. DEAE-52-cellulose and DEAE-Sepharose Fast Flow columns are the primary two anion exchange columns used to separate the neutral and acidic polysaccharide fractions. For instance, crude PSP was subjected to a DEAE-52-cellulose chromatography column (Ø2.5 × 40 cm) with elution of distilled water and 0.05 M NaCl solution in sequence, then a neutral polysaccharide was obtained; after further purification of Sephacryl S-100 column (Ø1.6 × 100 cm) with elution of distilled water, two neutral fractions composed of galactose (Gal), glucose (Glc), and fructose (Fru) were separated from that neutral polysaccharide (15).

However, on the whole, the extraction and purification methods of PSPs are limited in lab scale and lack advance extraction technology, such as microwave/ultrasonic-assisted extraction (32–35), subcritical water extraction (36), pulsed electric field-assisted extraction (37, 38), aqueous two-phase extraction (39), and membrane separation technology (40). Besides, there is no report on the large-scale or industrialization of PSPs. To achieve the standardized preparation and broaden the product market of PSPs in future, more easy-to-scale technologies such as ultrafiltration membrane, CTAB, and salting-out method should be used and optimized.

## Physicochemical and structural features

According to recent research, at least 37 polysaccharides have been isolated from the rhizome of *P. sibiricum*. The physicochemical and structural features of PSPs primarily including the molecular weight (Mw), monosaccharide composition, glycosidic bonds, and sequence of sugar residues are summarized in Table 1. It is seen that PSPs have a wide Mw range of 2.2–4,400 kDa, reflecting a huge difference in molecule size and property. These Mw data of PSPs were all detected by high-performance gel permeation chromatography (HPGPC) (2, 5, 14, 15, 41–44), while this method can only provide approximate Mw information. By the HPGPC method, the Mws of PSPs were calculated based on the linear dextran standards (0.18–2,000 kDa) calibration, but different standards significantly affected the Mw results (45). The conformation difference between PSPs samples and dextran would further increase this inaccuracy, particularly since that PSPs have anionic groups while dextran is neutral glucan. It is suggested that high-performance size-exclusion chromatography/field-flow fractionation coupled with multi-angle laser light scattering method should be used more in Mw analysis of PSPs, which can provide the absolute Mw, molecular size, and solution conformation (45, 46), therefore are essential in building the quality standard of PSPs in future.

**Figure 3:**
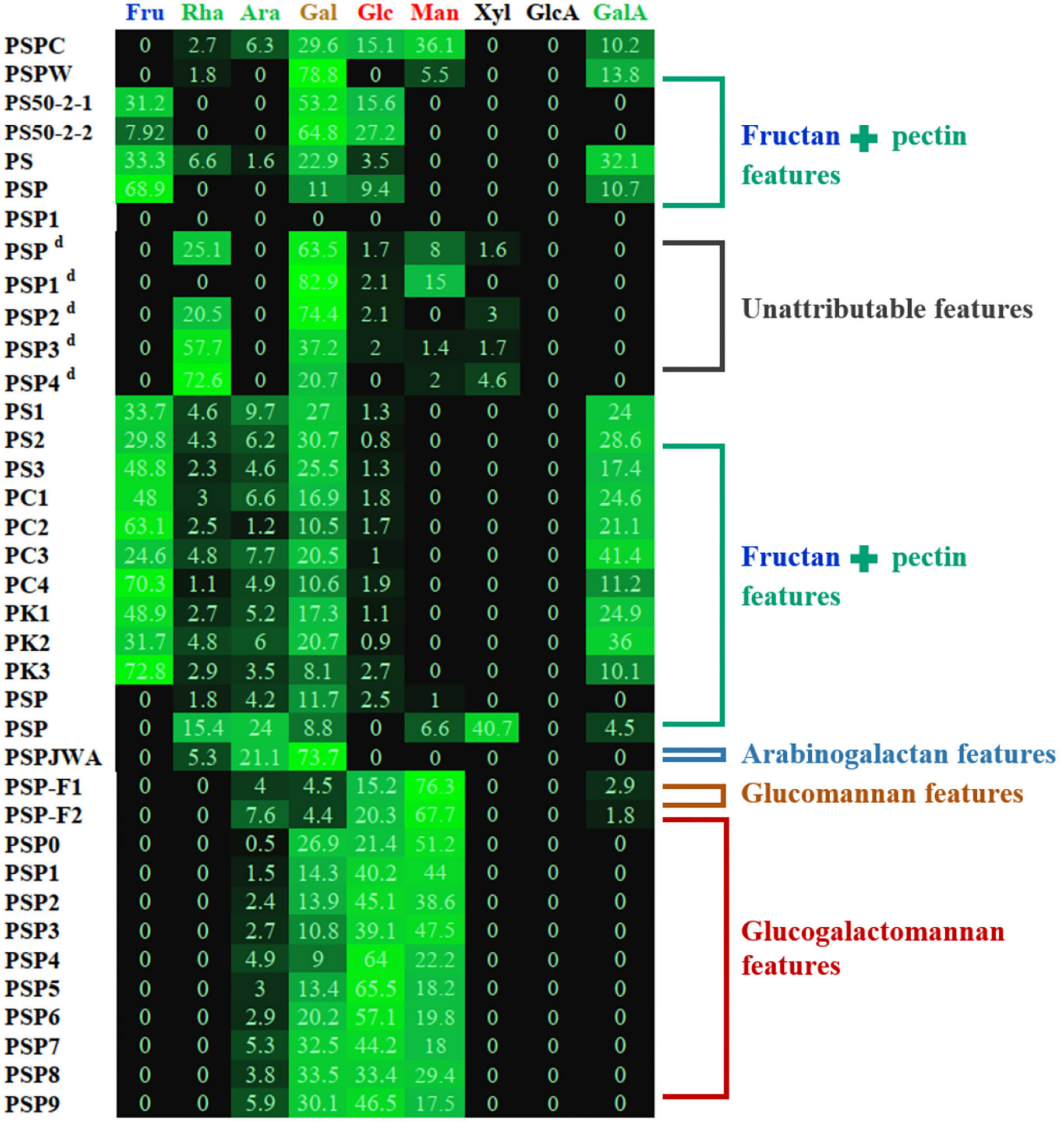
Heat map of monosaccharide of PSP based on summarized data (as shown in Table 1). ^d^: The determination of uronic acids (GalA and GlcA) was not mentioned in original papers, and the monosaccharide composition data were possibly calculated except for the uronic acids. Other details can be found in Table 1.

It can be concluded that polysaccharides from *P. sibiricum* are mostly pectins, fructans, and glucogalactomannan based on numerous studies (Table 1 and Figure 3). This conclusion differs from another review on PSP (11), in which glucogalactomannan is not included in common PSPs. The structural features of PSP are mainly elucidated using a series of chemical methods and techniques including Fourier transform infrared spectroscopy (FT-IR), high-performance liquid chromatography (HPLC), acid hydrolysis combining with high-performance anion exchange chromatography with pulsed amperometric detection (HPAEC-PAD), methylation analysis combining with gas chromatography-mass spectrometry (GC-MS), and nuclear magnetic resonance (NMR).

However, the structure analysis of fructan and pectin of PSPs should be cautious, since fructan is relatively not stable in acid degradation (47) and pectin is acid-resistant, which therefore will make the data of monosaccharide and glycosidic bond ratios inaccurate. For example, Amicucci et al. (48) found that the acid degradation of fructan inulin would produce fructose and also some non-monosaccharide side products, which affected the monosaccharide data. Moreover, during the methylation experiment of fructans, the fructose residues will convert into the corresponding partially methylated glucitol and partially methylated mannan alcohols due to the acid hydrolysis and the reduction of sodium borodeuteride (49). As a result, the glycosidic bonds of PSP fructan should be comprehensively combined with monosaccharide ratio, methylation analysis, and reliable references. For the pectin of PSPs, given the acid-resistance of α-1,4-GalA glycosidic bond (50), the methanolysis combined with the TFA hydrolysis method is preferred (51–53). Additionally, our previous study demonstrated that a weak acid hydrolysis condition (100°C, 2 M H_2_SO_4_, 0.5 h) could decrease the underestimation of the rhamnose (Rha) content of pectin compared to a stronger acid condition (100°C, 2 M H_2_SO_4_, 2 h) (54). Consequently, the optimization of the structure analysis of PSPs is sometimes necessary.

**Figure 4:**
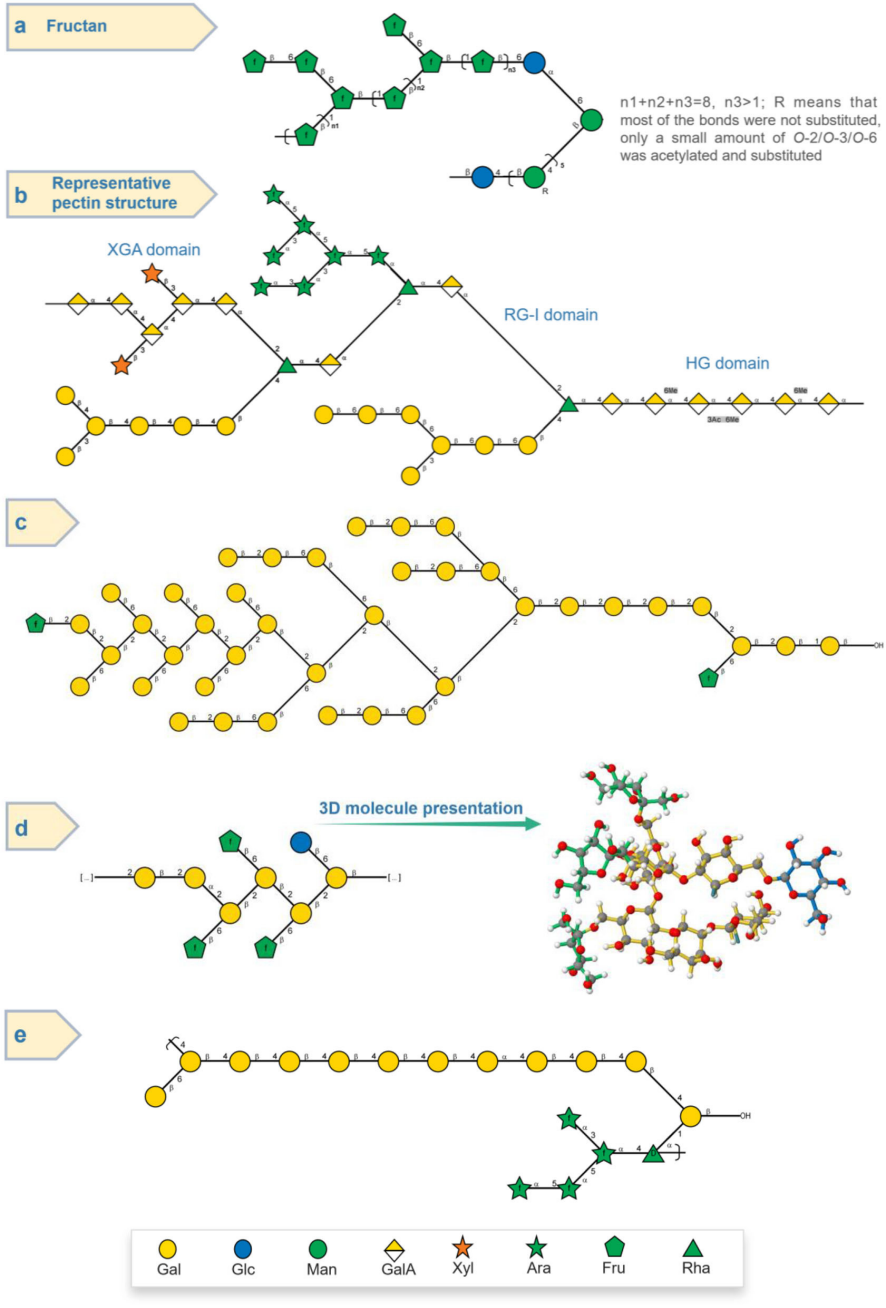
Structure presentation of different PSPs in Symbol Nomenclature for Glycans (SNFG) according to current studies. **(a)** Fructan-type PSP (23). **(b)** Representable pectin structure with homogalacturonan (HG), rhamnogalacturonan I (RG-I), and xylogalacturonan (XGA) domains. **(c,d)** Two novel PSPs (15). **(e)** Arabinogalactan-type PSP (14).

Wang et al. (23) used HPAEC-PAD, FT-IR, and methylation combined with GC-MS and 1D/2D NMR to reveal the structure of a novel polysaccharide from *P. sibiricum*. It was found that this POP had a main chain composed of → 1)-β-D-Fru*f*-(2 → 1)-β-D-Fru*f*-(2 → , → 1,6)-β-D-Fru*f*-(2 → 1)-β-D-Fru*f*-(2 → , and the side chain consisting of β-D-Fru*f*-(2 → , β-D-Fru*f*-(2 → 6)-β-D-Fru*f*-(2 → at *O*-6 positioned as shown Figure 4a. Importantly, the sugar chain mainly composed of → 1)-β-D-Glc*p*-(4 → and → 1)-β-D-Man*p*-(4 → was proposed to attach with the fructan region. The NMR signals at δ 1.90–2.10, 1.99/173.42, and 2.00/20.65 ppm regions were attributed to the *O*-acetyl of mannose (Man); furthermore, 2D NMR analysis indicated that the acetylated Man included → 4)-2-*O*-acetyl-β-D-Man*p*-(1 → , → 4)-3-*O*-acetyl-β-D-Man*p*-(1 → , and → 4)-6-*O*-acetyl-β-D-Man*p*-(1 → .

In addition to the fructan, the pectin of POPs was also the main polysaccharide. Zhao et al. (5) studied the saccharide mapping of more than 10 polysaccharides from three common *Polygonatum* spp. including *P. sibiricum* (PS), *P. cyrtonema* (PC), and *P. kingianum* (PK), respectively. It was proved that polysaccharides from PS, PC, and PK had pectic polysaccharides and fructans through specific enzymatic (pectinase, invertase, β-galactosidase, arabinase, etc.) treatments. While the polysaccharides from PS, PC, and PK consisted of not only fructans but also pectins, with a main Mw of more than 630 kDa. In addition, Gal, arabinose (Ara), Rha, and xylose (Xyl) might be present in the side chains of pectins, which indicated that pectin of PSPs possibly had the homogalacturonan and rhamnogalacturonan domains as shown in Figure 4b. Besides, the FT-IR absorption bands in 1,740 and 1,630 cm^−1^ indicated that the pectin of PSPs was esterified.

Two studies provided evidence for the existence of glucogalactomannan. One was from Sun et al. (22) and another was reported by Li et al. (20). The former revealed the structure of CPSP with high contents of Glc (15.1%), Gal (29.6%), and Man (36.1%). While the latter presented more significant monosaccharide composition features of glucogalactomannan of nine PSP fractions (Table 1) whose total amounts of Glc (9.0–33.5%), Gal (21.4–65.5%), and Man (17.5–51.2%) were more than 90%. These glucogalactomannan features can be observed in Figure 3. These monosaccharide composition features were similar to the previous report (12). Besides, their Mws were distributed between 4.4 and 75.3%. According to Ylitalo et al. (13), two glucomannan-type PSPs (F1 and F2) were also found in *P. sibiricum*. PSP-F1 was composed of Ara (4.0%), Man (76.3%), Glc (15.2%), Gal (4.5%), and PSP-F2 consisted of Ara (7.6%), Man (67.7%), Glc (20.3%), and Gal (4.4%).

Additionally, an arabinogalactan-type PSP with Gal, Ara, and Rha in a ratio of 14:4:1 was also isolated from *P. sibiricum*, as shown in Figure 4e. After methylation experiment and 2D NMR spectra elucidation, PSP was found to have the main chain composed of → 4)-Gal*p*-(1 → (59.2%) and some possible side chain units including Gal*p*-(1 → (7.8%), → 4,6)-Gal*p*-(1 → (6.2%), → 3,6)-Gal*p*-(1 → (2.7%), Ara*f*-(1 → (6.6%), → 5)-Ara*f*-(1 → (7.4%), and → 3,5)-Ara*f*-(1 → (5.0%). This arabinogalactan-type polysaccharide with a 1,4-galactan backbone was different with arabinogalactan from *Cynanchum atratum* (55) and *Carthamus tinctorius* L. (with 1,3-galactan backbone) (56), and banana (57).

Recently, two polysaccharides (PSP50-2-1 and PSP50-2-2) with novel structures from the rhizome of *P. sibiricum* were studied (15). The low content of Fru and the absence of GalA of PSP50-2-1 and PSP50-2-2 proved that they were not the common fructan- and pectin-type polysaccharides, while their relatively high contents of Gal (53.2, 64.8%) and Glc (15.6, 27.2%) were also unique features. As shown in Figure 4d, based on methylation and 2D NMR analysis, PSP50-2-1 was found to be composed of β-D-Glc*p*-(1 → , → 2)-β-D-Gal*p*-(1 → , → 2,6)-β-D-Gal*p*-(1 → , and β-D-Fru*f*-(2 → with a molar ratio of 1.0:1.9:4.1:3.0, respectively; and PSP50-2-2 was composed of β-D-Glc*p*-(1 → , β-D-Gal*p*-(1 → , → 2)-β-D-Gal*p*-(1 → , → 6)-β-D-Gal*p*-(1 → , → 2,6)-β-D-Gal*p*-(1 → , and β-D-Fru*f*-(2 → with a molar ratio of 9.7:1.0:9.6:3.8:10.7:2.1, respectively, as shown in Figure 4c. Apart from common fructan and pectin, there was also possible (arabino)xylan found by Luo et al. (27). It was found that the purified PSP consisted of Man, Rha, GalA, Glc, Xyl, and Ara with the molar ratio of 6.6:15.4:4.5:8.8:40.7:24.0, respectively. The high ratio of Xyl indicated the possible existence of xylan, while some Rha, GalA, and Ara might belong to the pectin region (46, 54, 58).

Notably, some data should be selectively considered in describing PSP features. For instance, Sun et al. (22) compared two water-extracted polysaccharides (PSPC and PSPW) from crude and wine-processed *P. sibiricum*, respectively, and found that PSPC consisted of 83.3% of neutral sugar and 9.4% of uronic acid, and PSPW contained 41.8% of neutral sugar and 20.1% of GalA. However, these data were not consistent with their monosaccharide composition, particularly that PSPW consisted of 85.2% (mass ratio) of neutral sugar (Rha, Ara, Man, Glc, and Gal) and 14.8% of uronic acid (GalA). This inconsistency of neutral sugar and uronic acid contents may be related to the unstable monosaccharide analysis method (53), which should be paid more attention to, otherwise will be misleading data.

## Biosynthesis of polysaccharide

The study of the molecular mechanism of PSP biosynthesis can help understand the PSP at the molecular level and may benefit modulating the yield and properties of PSPs in future large-scale production. Some researchers only focused on the fructan biosynthesis of PSPs (53); while the latest research by Feng et al. (59) revealed both fructan and pectic polysaccharides of PSPs, and it was discussed here. Specifically, Feng et al. (59) found 17 candidate genes associated with the polysaccharide content, which were considered to involve PSP biosynthesis in *P. sibiricum*. Sucrose is converted to D-Glc-6-phosphate (D-Glc-6P), D-Fru, and D-Glc. Then, there are two directions for the pathway based on the D-Glc-6P: one is the conversion of D-Glc-6P to α-D-Glc-1P by phosphoglucomutase, another is the conversion of D-Glc-6P to D-Fru-6P by Glc-6P isomerase isomerization. Thereafter, most verified enzymes such as Glc-1P adenylyltransferase and UDP-Glc 4-epimerase take part in the biosynthesis. Besides, UDP-Glc-6-dehydrogenase and UDP-GlcA 4-epimerase can accomplish the conversion among UDP-Glc, UDP-GlcA, and UDP-GalA. More details of apiose, Xyl, Man, Rha, Ara, and Gal can be found in the research paper of Feng et al. (59). At last, the activated sugar units are assembled into growing polysaccharide chains by various glycosyltransferases (GTs). Apart from the main fructan and pectin polysaccharides, other types of polysaccharides containing Glc, Gal, etc. should be further explored on their biosynthesis.

## Biological activities

**Figure 5:**
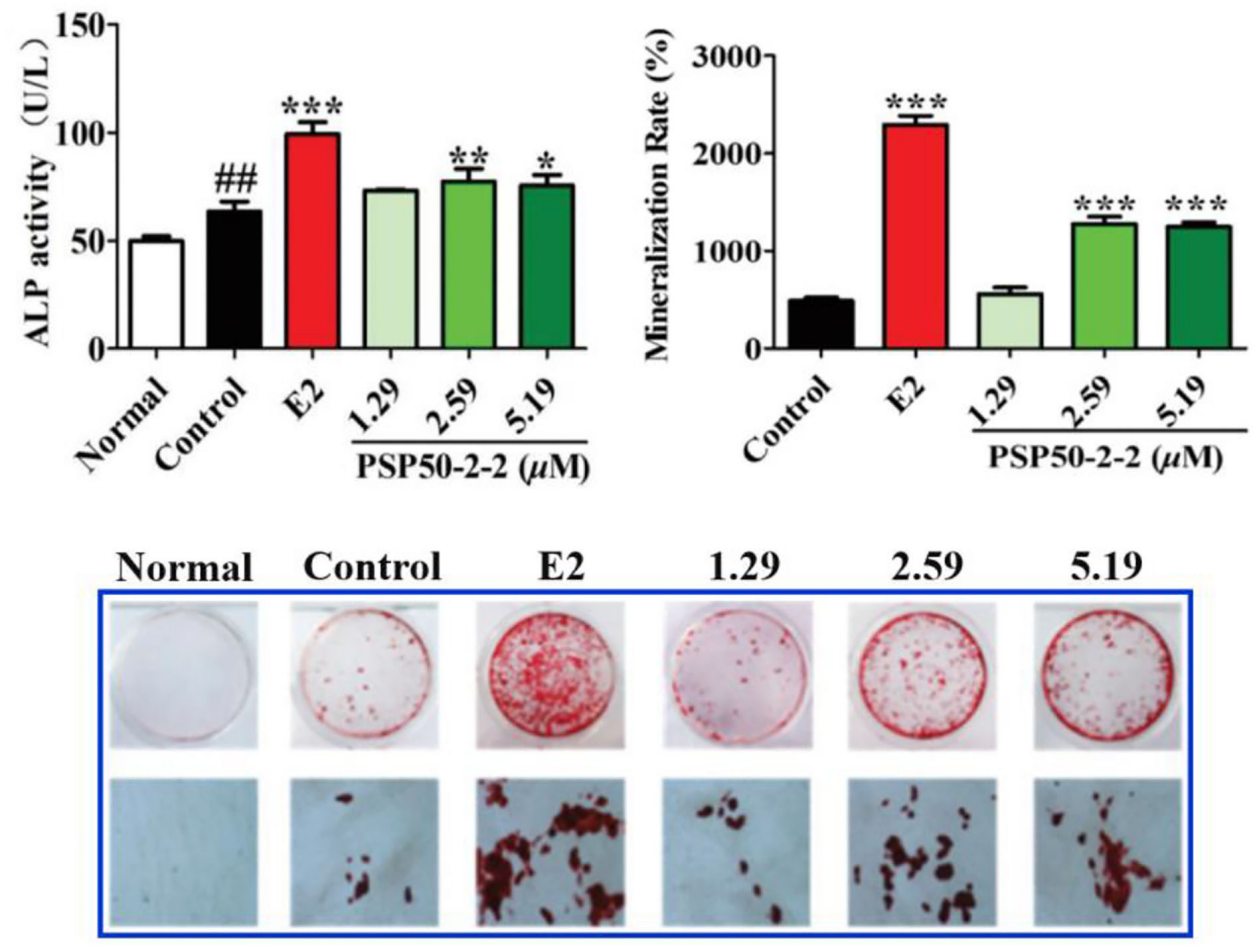
Effects of PSP50-2-2 fraction on the mineralization of MC3T3-E1 cells evidenced by the ALP activity, mineralization rate, and image of MC3T3-E1 cells. Images show alizarin red S staining of MC3T3-E1 cells treated with different concentrations of PSP50-2-2 (15). Copyright 2021, Royal Society of Chemistry.

**Figure 6:**
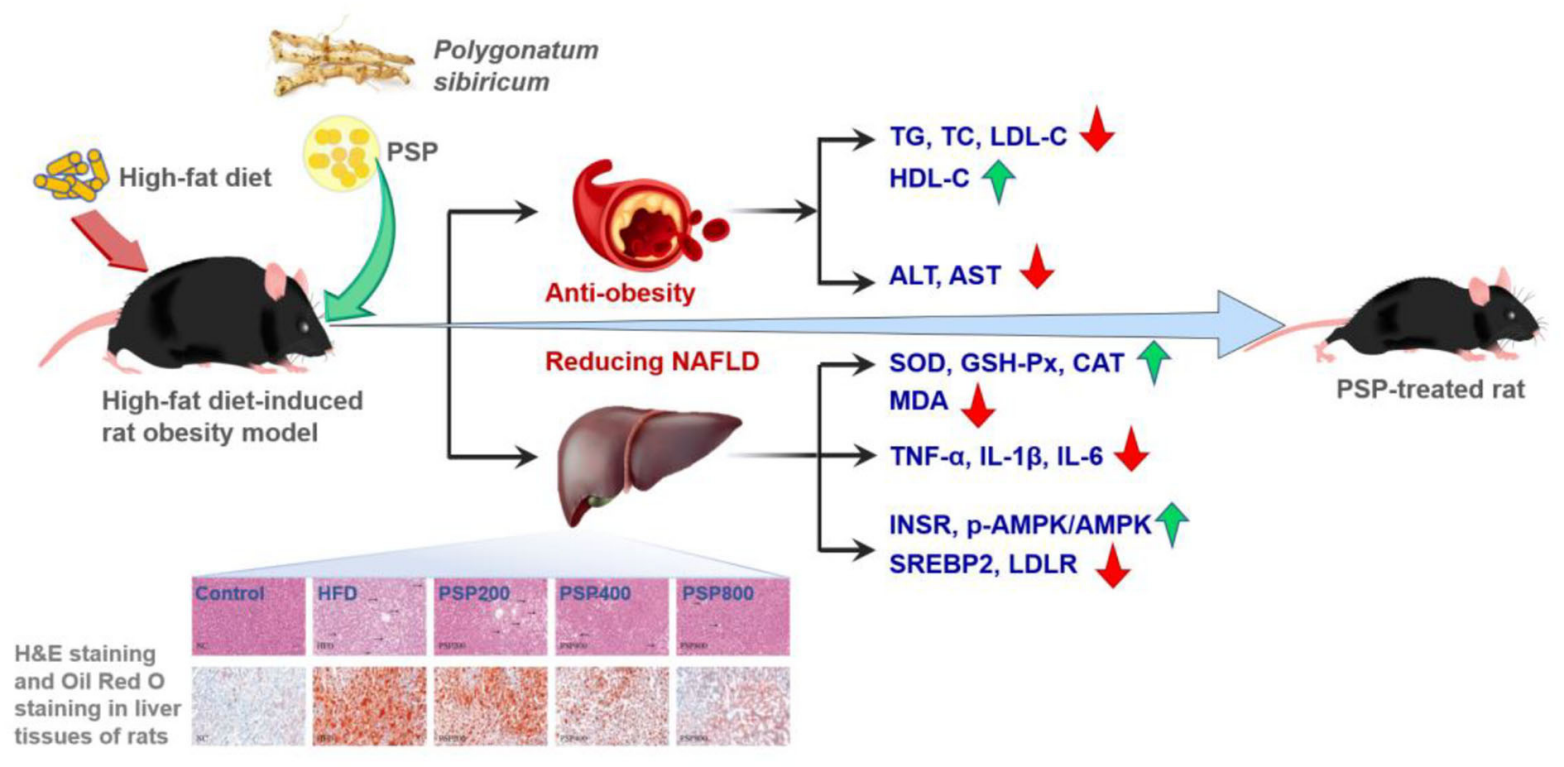
Possible mechanism by which PSPs confer weight loss and alleviate non-alcoholic fatty liver disease (NAFLD) in rats with HFD-induced obesity (18). Copyright 2022, Elsevier.

**Figure 7:**
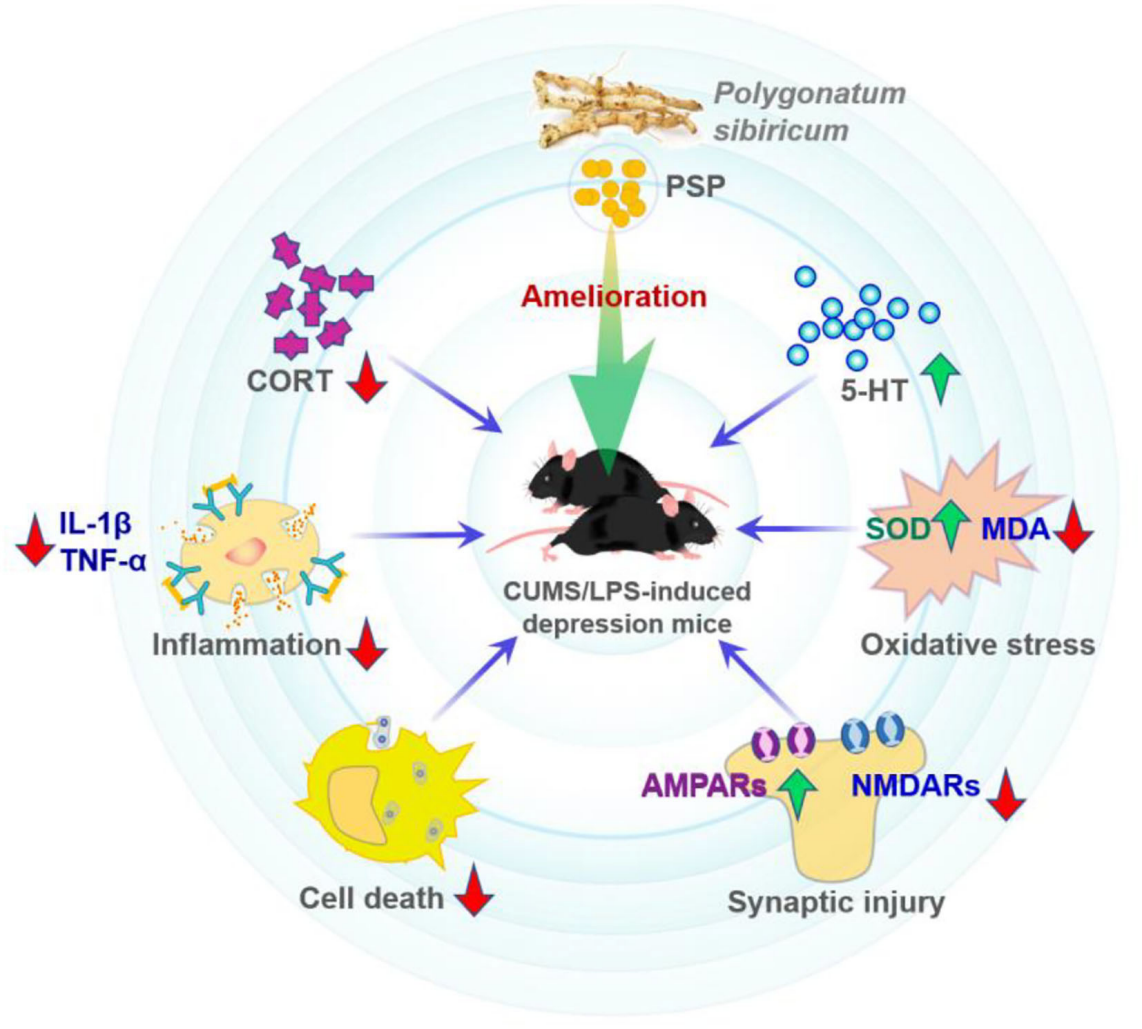
Possible amelioration mechanisms of depression-like behaviors by PSP (21).

PSPs have been widely explored for their various bioactivities, including immunomodulatory, osteogenic, anti-obesity, anti-diabetes, anti-depression, antioxidant, and anti-aging activities. The corresponding bioactivity effect and mechanism have been summarized in Table 2. Also, some key results and regulation pathways are presented in Figures 5–**7**, respectively.

**Table 2:** Representative bioactivities of *Polygonatum sibiricum* polysaccharides.

**Biological activities**	**Polysaccharide name**	***In vitro/*** ***in vivo***	**Model system**	**Doses/duration**	**Effects/mechanism**	**Reference**
***Antioxidant***						
	PSP	*In vitro*	Assay	0.1, 0.2, 0.4, 0.6, 0.8, 1.0, 2.0, 3.0, 4.0, and 5.0 mg/mL, respectively	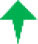 Scavenging IC_50_ rate of DPPH radical (3.07 mg/mL), ABTS (0.68 mg/mL), hydroxyl radical (1.04 mg/mL)	Bai et al. (41)
	PSP1	*In vitro*	Assay	0.5, 1.0, 1.5, and 2.0 mg/mL, respectively	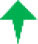 Scavenging rate of DPPH, hydroxyl, superoxide radicals of PSP1 (2.0 mg/mL) were 56.3, 57.0, and 41.7%, and ABTS of PSP1 (2.0 mg/mL) was 33.3%	Wang et al. (23)
***Anti-obesity***	PSP	*In vivo*	High-fat diet (HFD)-induced rat obesity model	HFD group: rat fed a HFD diet (63.6% normal chow, 20.0% sucrose, 15.0% lard, 1.2% cholesterol, and 0.2% sodium cholate), 4 weeks Low/medium/high dose group PSP group: 200, 400 and 800 mg/kg/d, 6 weeks	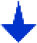 Body weight, serum total cholesterol, triglyceride, LDL-C levels, ALT, AST activity, MDA content, and levels of TNF-α, IL-1β, and IL-6; 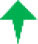 Serum HDL-C levels, hepatic SOD, catalase, and glutathione peroxidase activity	Zeng et al. (18)
***Anti-aging***	PSP	*In vivo*	D-Gal-induced heart aging mice	Model group: D-Gal 500 mg/kg/d for 60 days; Low-dose PSP group: 200 (PSP) + 500 (D-Gal) mg/kg/d for 60 days; High-dose PSP group: 400 (PSP) + 500 (D-Gal) mg/kg/d for 60 days	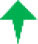 Cardiac troponin, creatine kinase, p21, and p53 levels; 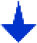 ROS & MDA levels; 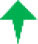 SOD level; 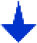 DNA damages and lipid peroxidation induced by oxidative stress (as indicated by reduced levels of 8-OHdG and 4-HNE	Ma et al. (60)
	PSP	*In vivo*	D-Gal-induced brain aging mice	Model group: D-Gal 50 mg/kg/d for 60 days; Low-dose PSP group: 200 (PSP) + 500 (D-Gal) mg/kg/d for 60 days; High-dose PSP group: 400 (PSP) + 500 (D-Gal) mg/kg/d for 60 days	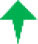 Cognitive function during brain aging 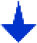 Escape latency time (*p* < 0.05) 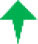 Number of times mice crossed the platform (*p* < 0.05). 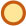 A total of 37, 13, and 679, circRNAs, miRNAs, and mRNAs, respectively, were significantly altered by PSP treatment.	Zhang et al. (61)
***Immunomodulatory***					
	PSPC\PSPW	*In vitro*	RAW 264.7 cells	12.5–200 μg/mL, 12 h	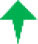 Cells viability, phagocytic capacity, acid phosphatase activity, NO production	Sun et al. (22)
		*In vivo*	Spleen deficiency male mice model	PSP 200, 400, and 800 mg/kg, respectively	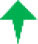 Immune functions; 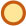 Reversing the decline of secretions of IL-2, IL-6, TNF-α, and IFN-γ to a normal range	
	PSP	*In vivo*	Cyclophosphamide (CY)-induced immunosuppressed chickens	Negative control group: regular diets + sterile saline; CY-induced group: regular diets + 80 mg/kg CY; PSP control group: regular diets + 800 mg/kg PSP + sterile saline; CY-induced + PSP group: regular diets + 800 mg/kg PSP + treated 80 mg/kg CY	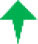 Weight, morphologic integrity and function of thymus and spleen; 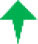 Antioxidant by increasing SOD level and 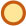 decreasing MDA level in serum; 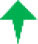 Proliferation of peripheral blood T lymphocytes; 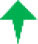 Stimulating serum immunoglobulin; 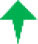 Levels of immune-related cytokines (IL-2, IL-6 and IFN-γ).	Shu et al. (62)
	PSP	*In vitro*	Splenocytes	100, 200, and 400 mg/mL, 48 h	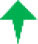 Proliferative responses of splenocytes;	Chen et al. (26)
		*In vivo*	CY-induced immunosuppressed mice	Positive control: levamisole hydrochloride 10 mg/kg; PSP-L group: 100 mg/kg; PSP-M group: 200 mg/kg; PSP-H group: 400 mg/kg; Negative control (CY); …	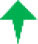 Expression of cytokines (TNF-α and IL-2); 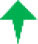 CD4^+^/CD8^+^ ratio T lymphocytes; 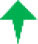 Phagocytosis of mononuclear macrophages; 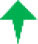 T/B lymphocyte proliferation in the spleen of mice	
	PSP1/PSP2/ PSP3/PSP4	*In vitro*	RAW 264.7 cells	0, 5, 10, 25, 50, 100, 200, and 400 μg/mL, 24 h	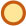 Nontoxic to macrophages (0–400 μg/mL); 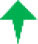 Phagocytosis of the RAW 264.7 cell line (effect order of PSP3>PSP2>PSP4>PSP1);	Wang et al. (25)
	PSP3	*In vivo*	CY-induced immunosuppressed mice	Positive group: 0.2 mL levamisole (10 mg/kg); PSP3 group: 100, 200, 400 mg/kg	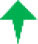 Restoring organ indexes; 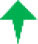 Proliferation of splenocytes and activity of NK cell; 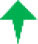 IL-2 and TNF-α, 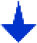 IL-4 and IL-10 levels	
***Gut microbiota regulation actvitiy***					
	PSP	*In vivo*	Mice	PSP group: PSP (200 mg/kg) once a day. Control group: the same volume of distilled water	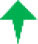 Levels of SCFAs including acetic acid, propionic acid, and butyric acid; 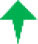 Relative abundance of *Firmicutes* and 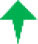 in *Bacteroidetes* at the phylum level; 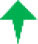 The abundance of *Lactobacillus*, 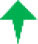 *Lachnospiraceae* and *Bacteroides* reduced (at the genus level)	Luo et al. (27)
***Osteogenic activity***					
	PSP50-2-1	*In vitro*	MC3T3-E1 cells	0.02, 0.1, and 0.5 μM, respectively, 48 h	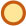 No effect on ALP activity of the MC3T3-E1 cells	Liu et al. (15)
	PSP50-2-2	*In vitro*	MC3T3-E1 cells	1.29, 2.59, and 5.19 μM, respectively, 48 h	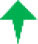 Differentiation and mineralization of MC3T3-E1 cells significantly at 2.59 and 5.19 μM	Liu et al. (15)
	PSP	*In vitro*	Bone marrow mesenchymal stem cells (BMSCs)	0, 5, 10, 25, and 50 mg/L PSPs, respectively, were added to the BMSCs	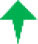 Osteogenic differentiation of BMMSCs	Zhao et al. (17)
	PSP	*In vitro*	BMSCs	200, 300, 400, and 500 mg/mL	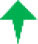 Proliferation of BMSCs during osteogenic differentiation; 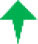 Expression of ALP, PINP, BMP-2, OCN, BSP and SPAR in osteoblasts	Zong et al. (7)
***Antiglycation***					
	PSP	*In vitro*	Assay	0.75, 1.50, and 3.00 mg/mL, respectively	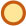 Presenting the strongest inhibitory activity (30.2%) at 3 mg/mL with AGEs	Zhao et al. (2)

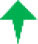
 increase, 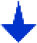 decrease, 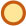 other phenomena or trends.

### Immunomodulatory activity

Numerous experiments have shown that immunostimulatory polysaccharides can interact with the immune system, which can trigger some cellular/molecular events, thereby activating the immune system (63). PSPs are also the immunostimulatory polysaccharides that have been verified mainly by both cell (RAW264.7 or splenocytes) and animal experiments of cyclophosphamide (CY)-induced immunosuppressed mice model. For example, Chen et al. (26) investigated the immunomodulatory activity of PSP through *in vitro* and *in vivo* experiments. In the *in vitro* splenocytes experiments, PSP with different concentrations (100, 200, and 400 mg/mL) increased the proliferation of splenocytes and the phagocytosis of mononuclear macrophages. On the other hand, PSP could recover the body weight of CY-treated mice and increase the CD4^+^/CD8^+^ ratio in a dose-dependent manner, as well as improve TNF-α and IL-2 in serum. Also, PSP was found to accelerate the recovery of natural killer cell activity (26). Additionally, in the cyclophosphamide-induced immunosuppressed chickens (62), it was found that PSP with a dose of 800 mg/kg significantly (1) promoted the weight, morphologic integrity, and function of immune organs (thymus and spleen), (2) improved the antioxidant by increasing SOD level and decreasing MDA level in serum, (3) stimulated serum immunoglobulin and improved the proliferation of peripheral blood T lymphocytes, and (4) upregulated the mRNA expression levels of immune-related cytokines (IL-2, IL-6, and IFN-γ).

Consistent with the above results, Wang et al. (25) recently studied *in vitro* immunoregulatory activity of four PSP fractions (PSP1/PSP2/PSP3/PSP4) through RAW 264.7 cells experiment, and results suggested that they were nontoxic to macrophages (0–400 μg/mL) and had improved the phagocytosis of the RAW 264.7 cell line with positive effect order of PSP3>PSP2>PSP4>PSP1. Consequently, PSP3 was further subjected to CY-induced immunosuppressed mice experiment, and it was found that PSP3 could significantly restore the organ indexes, improve the proliferation of splenocytes and activity of NK cells, enhance the levels of IL-2 and TNF-α, and reduce the levels of IL-4 and IL-10 (25). Similarly, the crude PSP (CPSP) and wine-processed PSP (WPSP, prepared by mixing CPSP with rice wine) were also found to enhance the phagocytic capacity, acid phosphatase activity, and NO production of RAW264.7 cells (22). Moreover, both CPSP and WPSP enhanced the immune functions of the immunosuppressive model for spleen-deficient mice and increased the levels of IL-2, IL-6, TNF-α, and IFN-γ. However, the WPSP showed better immunological activities than CPSP, which might be due to their different structural features such as different monosaccharide compositions as summarized in Table 1. Additionally, the cetyltrimethylammonium bromide (CTAB)-modified PSP was applied to prepare ovalbumin-absorbed CTAB-modified PSP cubosomes, which was also found to promote the lymphocyte proliferation, increase the ratio of CD4^+^/CD8^+^ T cells, and activate the dendritic cells in lymph nodes (64). These *in vitro* and *in vivo* findings demonstrate that PSPs have immunomodulatory activity and the potential to be one immunostimulant.

### Osteogenic activity

Osteoporosis is one common degenerative bone disease, and the bone loss in osteoporosis can be reduced by recovering the osteogenic capacities of bone marrow-derived mesenchymal stem cells (BMSCs) (65). Studies show that PSPs have anti-osteoporotic effect by promoting osteogenic differentiation or/and the apoptosis of myeloma cells. For instance, Zhao et al. (17) investigated the effect of PSPs treatment on the osteogenic differentiation of BMSCs. Results suggested that PSPs with different concentrations (5, 10, 25, and 50 mg/L) enhanced the osteogenic differentiation of BMMSCs by improving levels of alkaline phosphatase (ALP) and Runt-related transcription on factor-2 expression, Recombinant Collagen Type I Alpha 1 and osteocalcin (OCN), and increasing the formation of a mineralized nodule. Notably, 25 mg/L PSP treatment exhibited the greatest enhancement of osteogenic activity. These results indicated that PSP could increase the osteogenic differentiation of BMSCs and had the treatment potential for patients with myeloma *via* the regulation of phosphoinositide 3-kinase (PI3K)/protein kinase B (PKB)/mammalian target of rapamycin (mTOR) signal pathway (17). Similarly, Zong et al. (17) revealed that PSPs promoted the proliferation of BMSCs during osteogenic differentiation, and increased the expression of ALP, procollagen type I N-terminal propeptide (PINP), bone morphogenetic protein-2 (BMP-2), OCN, bone sialoprotein (BSP), and SPARC in osteoblasts. Furthermore, Liu et al. (15) demonstrated that a purified fraction PSP50-2-2 (2.59 and 5.19 μM) promoted the differentiation and mineralization of MC3T3-E1 cells *in vitro* (Figure 5). In addition, the polysaccharide-rich extract from *P. sibiricum* was found to decrease the loss of bone marrow hematopoietic stem and progenitor cells and common lymphoid progenitors suppressed by triple-negative breast cancer tumor. The above findings well-described the close relationship and some regulation pathways between PSP and osteogenic activity; however, research on deeper mechanisms and more clinical experiments should be further conducted.

### Anti-obesity and anti-diabetes

Obesity, one of the common chronic diseases, is increasing rapidly worldwide, causing global public health concerns. PSPs have been found to possess anti-obesity and anti-diabetes effects (18, 19). For instance (as shown in Figure 6), with the administration of PSPs at doses of 200, 400, and 800 mg/kg/d, respectively, the high-fat diet-induced rat with obesity showed a decrease in body weight, contents of serum total cholesterol, low-density lipoprotein cholesterol (LDL-C) and hepatic malondialdehyde, activities of hepatic aspartate aminotransferase and alanine aminotransferase, and hepatic levels of inflammatory cytokines, and also the increase of serum high-density lipoprotein cholesterol (HDL-C), hepatic superoxide dismutase (SOD) level, activities of catalase, and glutathione peroxidase (18). Apart from the decrease in obesity, PSPs also ameliorated the rat's non-alcoholic fatty liver disease (NAFLD), which was featured by liver steatosis that occurred without an alcohol consumption history (66). PSP could promote lipid metabolism and decrease inflammation and oxidative stress. These effects were evidenced by the downregulated expression of sterol regulatory element-binding protein 2 and LDL receptor and the promoted phosphorylation of adenosine monophosphate-activated protein kinase, as well as the upregulated insulin receptor expression. These findings demonstrated that PSPs possess the possible application in reducing obesity and NAFLD (18).

Additionally, obesity is one of the most significant modifiable risk factors for type 2 diabetes prevention (67). Type 2 diabetes is a metabolic disorder characterized by chronic hyperglycemia and insufficient insulin secretion (68). In addition, long-term high blood glucose can cause various complications, such as diabetic retinopathy, which further increases the inconvenience of life. In an animal experiment (19), streptozocin-induced diabetes was used to evaluate the anti-diabetes effect of PSP intervention at doses of 200, 400, and 800 mg/kg/d, respectively. Results showed that PSP might decrease diabetic retinal injury by lowering blood glucose, inhibiting pathological angiogenesis, and preventing cellular apoptosis through the downregulation of signaling of Bax, EGF, p38, VEGF, and TGF-b and upregulation of Bcl-2. This result indicated that PSP supplementation might be an alternative for the prevention of diabetic retinopathy (19).

### Anti-depression

Cell and animal experiments showed that some polysaccharides possess anti-depressive effects (69, 70). Depression is one common neuropsychiatric disease that is featured by pleasure loss, behavioral despair, and sometimes suicidal thoughts or behaviors (21, 69). In one study (21), lipopolysaccharide (LPS) and chronic unpredictable mild stress were used to build the depression mice models to test the anti-depressive effects of PSP. As shown in Figure 7, it was found that PSP administration promoted the level of hippocampal 5-HT, inhibited the serum cortisol level and hippocampal oxidative stress, and decreased the IL-1β and TNF-α levels. Furthermore, PSP administration enhanced the hippocampal expression of p-Akt, pmTOR, GluA1, and GluA2; reduced the expression of caspase-3, GluN2A, and GluN2B; and prevented the damage of granular cells in dentate gyrus region. These findings demonstrated that PSP could ameliorate depression-like behaviors and synaptic and neuronal damage by possibly decreasing the oxidative stress/hypothalamic-pituitary-adrenal axis hyperfunction and the inflammatory response (21).

### Antioxidant

Antioxidant test for PSPs mainly includes chemical assay and cell or animal experiments. These common chemical assays are scavenging free radical tests, such as DPPH, ABTS, and hydroxyl free radicals. For instance, Wang et al. (23) revealed that PSP (2.0 mg/mL) had the scavenging rate of DPPH, hydroxyl, and superoxide radicals of PSP1 (2.0 mg/mL), which were 56.3, 57.0, and 41.7%, and ABTS of PSP1 (2.0 mg/mL) was 33.3%. Li et al. (20) used a steaming process (121°C, 0.12 MPa, 60 min) in PSP extraction, and it was found that with the increase of steaming treatment times, the IC_50_ of DPPH radical scavenging activity decreased from 3.3 to 0.1 mg/mL (PSP1–PSP9) and IC_50_ of ABTS radical scavenging activity decreased from 8.82 to 0.41 mg/mL (IC_50_ of 0.41 mg/mL of PSP9 was superior to Vc), while IC_50_ of hydroxyl radical scavenging activity of PSP was not strong (20). There was also the antioxidant test of PSP *in vivo*. In a heart aging mice model induced by D-Gal (500 mg/kg/d), PSP from *P. sibiricum* at doses of 200 and 400 mg/kg/d significantly reduced ROS and MDA, and increased SOD level, which suggested the PSP's antioxidant activity *in vivo* (60). Furthermore, PSP could reduce the DNA damage and lipid peroxidation induced by oxidative stress after oral administration (60). Other data related to the increase of SOD level and the decrease of MDA level by PSP oral administration were also reported (18, 60, 62).

### Other bioactivities

PSPs have also been revealed to have anti-aging, antiglycation activity, protective effect against neurotoxicity, and gut microbiota regulating activity. Ma et al. (60) and Zhang et al. (61), respectively, built the D-Gal-induced heart aging mice and D-Gal-induced brain aging mice models to evaluate the anti-aging effect of PSP. Results showed that PSP had a cardioprotective effect on D-Gal-induced heart-aging mice by inhibiting DNA damage and lipid peroxidation caused by oxidative stress (60). Moreover, Zhang et al. proved that PSP effectively ameliorated the cognitive dysfunction of mice during brain aging (61). The antiglycation effect of PSP was tested by antiglycation assay in the BSA-glucose model, which indicated that pectins in PSP had stronger inhibition on AGEs formation than fructans (2). Many other cases are reporting the antiglycation effect of pectin (71–73). In addition, as dietary fiber, PSPs were found to affect the composition of gut microbiota, as evidenced by the increase in the abundance of *Lactobacillus* and the decrease of *Lachnospiraceae* and *Bacteroides* after PSPs treatment (27).

## Conclusion and perspectives

PSPs are one of the main bioactive compounds for the multiple healthy functions of *P. sibiricum*, which have antioxidant, osteogenic activity, anti-obesity, anti-diabetes, anti-depression, antiglycation, protective effect against neurotoxicity, and gut microbiota regulating activity. It is concluded that PSPs are primarily prepared by hot-water extraction and purified by the Sevag method combined with dialysis, also there are some cases with finer purifications using ion exchange or gel column chromatography; while the PSPs yield decreases with the increase of purification fineness and number of process. Furthermore, PSPs are revealed to consist of different structural polysaccharides, in which pectin and fructan are the two most common polysaccharides, and others include galactomannan, glucomannans, and arabinogalactan-type polysaccharides. In addition, the amount and structural composition of PSPs can be the key remarks in distinguishing the *P. sibiricum* quality. In summary, PSPs are normally a polysaccharide mixture with different structural features and multiple bioactivates, which possess huge potential in the application of functional food and medicine fields.

Nevertheless, there are still many challenges that need to be addressed. (1) The extraction and purification methods of PSPs are limited in lab scale and with a relatively low yield of PSPs; therefore, some large-scale and environmentally friendly methods should be explored for PSPs preparation in future. (2) Preparation process significantly affects the yield, structural features, and even bioactivities of PSPs, while the detailed relationships between preparation conditions and PSPs properties are required for further revelation. (3) Currently, PSPs content and structure in *P. sibiricum* material in different reports are significantly different, suggesting the lack of a comprehensive quality standard for PSPs, and consequently, more efforts should be paid to build the quality standard of PSPs. (4) Structural study of PSPs has mainly focused on the molecular weight, monosaccharide composition, and glycosidic linkages, while their advanced structural features require to be uncovered through SEC-MALLS, atom force microscope, and molecular simulation technology. (5) The relationship between the complex structural features and multiple bioactivities of PSPs needs to be discovered deeply; for example, the ratio of pectin/fructan in PSP was found to affect its antiglycation effect, while the internal mechanism was still unknown. (6) Given that most bioactive effects of PSPs have been measured *in vitro*, more animal studies and clinical studies should be conducted to better explain the mechanisms and “more realistic” effects of PSPs on human health. (7) Nowadays, the structure and bioactive information of PSPs have been explored extensively; however, their applications as functional food components are still insufficient; and how to strengthen the application of PSPs should be paid more attention and effort in future.

## Author contributions

Writing-original draft and conceptualization: DL and WT. Conceptualization and supervision: CH and SN. All authors have read and agreed to the published version of the manuscript.

## Funding

This work was supported by funding provided by the Scientific Research Startup Foundation for Introducing Talent of Zhejiang Shuren University, China (2021R033), the Open Project Program of State Key Laboratory of Food Science and Technology, the Nanchang University (SKLF-KF-202210), and the Scientific Research Startup Foundation for Introducing Talent of Zhejiang University of Technology, China (2021133013929).

## Conflict of interest

The authors declare that the research was conducted in the absence of any commercial or financial relationships that could be construed as a potential conflict of interest.

## Publisher's note

All claims expressed in this article are solely those of the authors and do not necessarily represent those of their affiliated organizations, or those of the publisher, the editors and the reviewers. Any product that may be evaluated in this article, or claim that may be made by its manufacturer, is not guaranteed or endorsed by the publisher.

## Abbreviations

NF-κB: nuclear factor kappa-light-chain-enhancer of activated B cells
p-ERK;: phosphorylated extracellular signal-regulated kinase
GFAP: glial fibrillary acidic protein
p-Akt: phosphorylated protein kinase B
mTOR: phosphorylation of the mammalian target of rapamycin
HPA: hypothalamic–pituitary–adrenal
LDL-C: low-density lipoprotein cholesterol
HDL-C: high-density lipoprotein cholesterol
NAFLD: non-alcoholic fatty liver disease
LDLR: low-density lipoprotein receptor
TNF-α: tumor necrosis factor-α
IL-1β: interleukin-1β
IL-6: interleukin-6β
AMPK: adenosine monophosphate activated protein kinase
CAT: catalase
ALT: alanine aminotransferase
AST: aspartate aminotransferase
SOD: superoxide dismutase
MDA: malondialdehyde
GSH-Px: glutathione peroxidase
ROS: reactive oxygen species
8-OHdG: 8-hydroxydeoxyguanosine)
4-HNE: 4-hydroxy-2-nonenal
PINP: procollagen type I N-terminal propeptide
BMP-2: bone morphogenetic protein-2
BSP: bone sialoprotein
LPS: lipopolysaccharide
CUMS: chronic unpredictable mild stress
CORT: cortisol
NMDARs: N-methyl-D-aspartate receptors
AMPARs: α-amino-3-hydroxy-5-methyl-4-isoxazolepropionate receptors
AGEs: advanced glycation end products
sacA;: β-fructofuranosidase
GPI;: Glucose-6-phosphate isomerase
AXS: UDP-apiose/xylose synthase
GMPP: Mannose-1-phosphate guanylyl transferase
UGDH: UDP-glucose 6-dehydrogenase
UGE: UDP-glucuronate 4-epimerase
RHM: UDP-glucose 4,6-dehydratase
GMDS: GDP-mannose 4,6-dehydratase
TSTA3: GDP-l-fucose synthase
GALE: UDP-glucose 4-epimerase
MEM: Mannan endo-1,4-beta-mannosidase
Bgal: β-galactosidase
Bglu: β-glucosidase
GPA: Glucose-1-phosphate adenylyltransferase
MPI: Mannose-6-phosphate isomerase
PMM: Phosphomannomutase.
